# Axial Myopia Is Associated with Visual Field Prognosis of Primary Open-Angle Glaucoma

**DOI:** 10.1371/journal.pone.0133189

**Published:** 2015-07-27

**Authors:** Chen Qiu, Shaohong Qian, Xinghuai Sun, Chuandi Zhou, Fanrong Meng

**Affiliations:** 1 Department of Ophthalmology, Eye and Ear, Nose, Throat Hospital, Shanghai Medical College, Fudan University, Shanghai, China; 2 State Key Laboratory of Medical Neurobiology, Institutes of Brain Science, Fudan University, Shanghai, China; 3 Key Laboratory of Myopia, Ministry of Health (Fudan University), Shanghai, China; 4 Shanghai Key Laboratory of Visual Impairment and Restoration (Fudan University), Shanghai, China; 5 Department of Ophthalmology, First People’s Hospital of Shanghai, Shanghai Jiaotong University, Shanghai, China; Massachusetts Eye & Ear Infirmary, Harvard Medical School, UNITED STATES

## Abstract

**Purpose:**

To identify whether myopia was associated with the visual field (VF) progression of primary open-angle glaucoma (POAG).

**Methods:**

A total of 270 eyes of 270 POAG followed up for more than 3 years with ≥9 reliable VFs by Octopus perimetry were retrospectively reviewed. Myopia was divided into: mild myopia (-2.99 diopter [D], 0), moderate myopia (-5.99, 3.00 D), marked myopia (-9.00, -6.00 D) and non-myopia (0 D or more). An annual change in the mean defect (MD) slope >0.22 dB/y and 0.30 dB/y was defined as fast progression, respectively. Logistic regression was performed to determine prognostic factors for VF progression.

**Results:**

For the cutoff threshold at 0.22 dB/y, logistic regression showed that vertical cup-to-disk ratio (VCDR; *p* = 0.004) and the extent of myopia (*p* = 0.002) were statistically significant. When logistic regression was repeated after excluding the extent of myopia, axial length (AL; *p* = 0.008, odds ratio [OR] = 0.796) reached significance, as did VCDR (*p* = 0.001). Compared to eyes with AL≤23 mm, the OR values were 0.334 (*p* = 0.059), 0.309 (*p* = 0.044), 0.266 (*p* = 0.019), 0.260 (*p* = 0.018), respectively, for 23 <AL≤24 mm, 24 <AL≤25 mm, 25 <AL ≤26 mm, and AL>26 mm. The significance of vertical cup-to-disk ratio of (*p* = 0.004) and the extent of myopia (*p* = 0.008) did not change for the cutoff threshold at 0.30dB/y.

**Conclusions:**

VCDR and myopia were associated with VF prognosis of POAG. Axial myopia may be a protective factor against VF progression.

## Introduction

The multifactorial and progressive nature of primary open-angle glaucoma (POAG) has been well established in regard to optic nerve damage and visual field (VF) loss. A broad spectrum of factors in glaucoma has been investigated in the past two decades. Intraocular pressure (IOP) is the main risk factor for glaucomatous optic neuropathy. A significant reduction in IOP is efficient in retarding disease progression, even in patients without high IOP [1]. Meanwhile, many other factors, such as disk haemorrhage [2] and systolic/diastolic blood pressure [3,4], have also been mentioned as contributing to POAG deterioration. However, clinical trials have yielded different results.

Whether myopic alterations affect glaucoma progression has not been asserted yet. In Asia, myopia prevalence has been increasing dramatically. Structural changes associated with myopia, including elongated axial length, tilt optic disk, parapapillary atrophy (PPA) and thinning of the lamina cribrosa and parapapillary sclera, may alter glaucoma susceptibility [5–8]. Many studies have not found myopia, usually examined in values of spherical equivalent, to have a significant role in VF progression [2,9–11]. However, some studies have identified certain extent of myopia as a potential prognostic factor [12–14].

To date, few studies have been conducted in China regarding the prognostic factors for POAG. Additionally, the extent of myopia and axial length were not regularly included as candidate parameters in previous studies. Therefore, the current study aimed to identify a rapidly deteriorating group of patients and to explore the potential risk factors that may forecast glaucomatous VF damage among these POAG patients, with a focus on myopia.

## Materials and Methods

### Patients and data collection

This retrospective study was approved by the Institutional Review Board and Ethics Committee of Eye, Ear, Nose and Throat (EENT) Hospital of Fudan University. The design and conduct of this study were in accordance with the principles of the Declaration of Helsinki. Written informed consent was obtained from each participant prior to enrolment. POAG patients who were diagnosed and followed up at the EENT Hospital of Fudan University from February 1998 to March 2014 were consecutively reviewed.

For all the patients, demographic data were obtained during the first visit. The baseline examinations included refractometry (Nidek ARK 700A, Gamagori, Japan), best-corrected visual acuity (BCVA; converted to a logarithm of the minimum angle of resolution of visual acuity, logMAR), slit lamp assisted biomicroscopy, gonioscopy, red-free fundus examination, Goldmann applanation tonometry, stereoscopic photographs of the optic disk (EOS D60 digital camera, Canon, Utsunomiyashi, Tochigiken, Japan), axial length (Sonomed A-1500, Lake Success, NY, USA), central corneal thickness (CCT) using ultrasonic pachymetry, and VF tests using Octopus perimetry (Octopus 101, Haag-Streit Inc., Koeniz, Switzerland). Two experienced ophthalmologists (X.S and S.Q) independently evaluated the masked stereoscopic photographs to check for the optic disk, vertical cup-to-disk ratio (VCDR), neural rim, major retinal vessel, and retinal nerve fiber layer (RNFL). Any disagreements were discussed; when agreements could not be reached, an additional ophthalmologist (F.M) was consulted. Myopia was classified into: mild myopia (-3.0 diopter [D] <myopia<0), moderate myopia (-6.0 D <myopia≤-3.0 D), marked myopia (-9.0 D ≤myopia≤-6.0 D), and non-myopia (0 D or more).

POAG was defined when patient had a normal open iridocorneal angle, glaucomatous optic neuropathy with notching, rim thinning, and RNFL defect, and corresponding VF defect (both high-tension and normal-tension patients). A minimal glaucomatous VF defect consisted of a cluster of ≥3, nonedge, contiguous points on the corrected probability plot, not crossing the horizontal meridian, with a probability of <5% in age-matched healthy controls (one of which was <1%). Under the circumstance that both of a given patient’s eyes were eligible, one eye was randomly selected.

Each patient had to be followed up for more than 3 years with no less than 9 reliable, compatible VFs and medically treated. All of them received a conventional course of medical therapy, in which a monotherapy was initially used and then a combination of topical hypotensive agents. If the glaucoma could not be controlled even with the maximum tolerated medical treatment, glaucoma surgery or laser treatment was recommended, which came to the endpoint of our follow up. Additionally, regular visits with follow-up intervals of no more than 6 months were required. Slit-lamp biomicroscopy, ophthalmoscope, BCVA and IOP had to be examined every time when patients visited the clinic. IOPs were measured every 1–3 month by Goldmann applanation tonometry during the office time. Optic disk sterophotographs and VF tests should be reexamined at 3 to 8-month intervals.

The exclusion criteria were refractive errors with spherical equivalent <-9.0 D or >6.0 D; any history of intraocular surgery or refractive surgery or laser therapy; BCVA less than 20/40, such as cataracts, that possibly influenced the visual acuity and VF tests; mean defect (MD) worse than 20dB; myopes with disk and fundus changes that may have impaired adequate evaluation of the optic nerve or VF for glaucoma; and concomitant other ocular diseases, such as optic disc anomalies, retinal diseases, or intracranial lesions.

### Visual field analysis

Taking into account the learning effect of the VF tests, the results of the first two tests were excluded. Only reliable (false positive/negative under 15% and reliability factor under 15) and compatible VF results were included. The change in MD over time, i.e., the MD slope, was used for evaluating visual progression. Based on previously reported rates of VF progression [15,16], two different threshold values (0.22 dB/y and 0.30 dB/y) were used to distinguish slow progression from fast progression. Initially, the median of the MD slope (0.22 dB/y) was set as the cutoff threshold. Eyes with MD slope >0.22 dB/y were defined as fast progression and those with MD slope ≤0.22 dB/y were classified into slow progression. Then 0.30 dB/y was set as the cutoff threshold. This meant eyes with MD slope >0.30 dB/y were fast progressed while those ≤ 0.30 dB/y were slow progressed.

### Statistical analysis

Statistical analysis was performed using SPSS (version 19.0, SPSS, Inc., Chicago, Illinois, USA) and SAS (version 9.2, SAS Institute, Inc., Cary, NC, USA) software. For univariate analysis, each parameter was compared between fast progression and slow progression using student t test, nonparametric analysis or χ^2^ test. Multivariate logistic regression analysis was performed when including all the candidate variables: age, gender, right/left eye, spherical equivalent (continuous variable), BCVA (logMAR), VCDR, CCT, axial length, baseline IOP, mean follow-up IOP, IOP reduction, IOP reduction percentage, initial MD value, follow-up period, and myopia extent (categorical variable; non-myopia was set as a reference). Taking into consideration the correlation between myopia and axial length, logistic regression was repeated, excluding the extents of myopia.

A probability value of <0.05 was defined for the statistical significance.

## Results

The characteristics of 270 eyes of the 270 POAG patients were listed in Table 1. The patients were 45.90 ± 15.29 years old with an initial MD of 6.209 ± 4.278 dB. The baseline IOP was 23.65 ± 5.54 mmHg. After following up for 5.605 ± 2.724 years (range 3.0–19.0 years), the mean follow-up IOP was 15.86 ± 2.45 mmHg and the median of MD the slope was 0.215 dB/y. Thus, 0.22 dB/y was first used to differentiate the fast and slow progression groups. Univariate analysis revealed that age, spherical equivalent, the extent of myopia, initial MD, BCVA (logMAR), VCDR, and axial length were significantly different between the two groups.

**Table 1:** Characteristics of patients with primary open-angle glaucoma. Fast/Slow progression was classified at the MD slope of 0.22 dB/y.

	POAG	Fast progression	Range	Slow progression	Range	p Value
Characteristics	(n = 270)	(n = 132)		(n = 138)		
Age at diagnosis (year)	45.90 ± 15.29	48.47 ± 16.25	18–84	43.45 ± 13.92	18–71	0.007^†^
Gender (male/female)	162/108	78/54		84/54		0.766^‡^
Subject eye (right/left)	114/156	53/79		61/77		0.500^‡^
Spherical equivalent (diopter)	-3.72 ± 2.98	-3.23 ± 3.23	-9.00–3.00	-4.18 ± 2.65	-9.00–4.50	0.010^†^
Extents of myopia						0.003^‡^
Non-myopia	53	36(66.1%)		17(33.9%)		
Mild myopia	48	29(62.0%)		19(38.0%)		
Moderate myopia	89	30(38.4%)		59(61.6%)		
Marked myopia	80	37(47.2%)		43(52.8%)		
BCVA(logMAR)	0.08 ± 0.10	0.09 ± 0.10	-0.08–0.03	0.07 ± 0.10	-0.18–0.30	0.020^†^
CCT (um)	545.11 ± 32.80	545.40 ± 33.93	470–619	544.83 ± 31.80	458–624	0.886^†^
Vertical cup-to-disk ratio	0.78 ± 0.14	0.81 ± 0.13	0.4–1.0	0.75 ± 0.14	0.3–1.0	<0.001^†^
Axial length(mm)	25.08 ± 1.51	24.82 ± 1.54	21.78–27.95	25.33 ± 1.44	21.26–29.25	0.005^†^
Baseline IOP (mmHg)	23.65 ± 5.54	24.06 ± 5.95	15–45	23.27 ± 5.12	14.5–40.0	0.242^†^
Mean follow-up IOP (mmHg)	15.86 ± 2.45	15.95 ± 2.42	9.0–22.0	15.78 ± 2.47	9.4–22.0	0.575^†^
IOP reduction (mmHg)	7.83 ± 4.81	8.17 ± 5.29	0.5–36.0	7.50 ± 4.30	0.1–24.0	0.251^†^
IOP reduction percentage (%)	31.10 ± 12.13	31.82 ± 12.07	2.94–83.72	30.42 ± 12.19	0.57–60.00	0.343^†^
Initial MD (decibels)	6.209 ± 4.278	6.826 ± 4.164	0.50–18.90	5.618 ± 4.317	0.10–18.60	0.020^†^
MD change (decibels)	1.620 ± 2.012	2.976 ± 2.065	0.70–13.90	0.323 ± 0.634	-2.00–2.30	<0.001^†^
Follow-up period (year)	5.605 ± 2.724	5.788 ± 2.757	3.0–19.0	5.430 ± 2.692	3.0–19.0	0.281^†^
MD slope (decibels/year)	0.284 ± 0.314	0.526 ± 0.267	0.222–1.738	0.052 ± 0.119	-0.375–0.217	<0.001^†^

POAG = primary open-angle glaucoma, BCVA(logMAR) = logarithm of the minimum angle of resolution of best-corrected visual acuity, CCT = central corneal thickness, IOP = intraocular pressure, MD = mean defect. Data are presented as number and mean ± SD or n(%), unless otherwise indicated.

^†^Student t test or nonparametric analysis;

^‡^ χ^2^ test

For the cutoff threshold at 0.22 dB/y, which was based on the median of the MD slope, multivariate logistic regression analysis was performed to screen for factors associated with VF progression. The candidate variables included age, gender, right/left eye, spherical equivalent (continuous variable), BCVA (logMAR), VCDR, CCT, axial length, baseline IOP, mean follow-up IOP, IOP reduction, IOP reduction percentage, initial MD value, follow-up period, and myopia extent. The results showed that VCDR (*p* = 0.004; odds ratio [OR] = 1.737 per 0.1 unit) and the extent of myopia (*p* = 0.002) were statistically significant (Table 2). The OR values of moderate myopia (*p* = 0.001) and marked myopia (*p* = 0.023) were 0.282 and 0.425, respectively, compared to non-myopia. In this study, we failed to find the association between VF progression and IOP, including baseline IOP, mean follow-up IOP, IOP reduction and IOP reduction percentage.

**Table 2:** Logistic regression analysis of factors associated with fast progression (MD slope >0.22 dB/y).

	OR (95% CI)	*p* Value
VCDR (per 0.1 larger)	1.737 (0.642–4.704)	0.004^†^
The extent of myopia		0.002^†^
Non-myopia	Reference	Not available
Mild myopia	0.796 (0.520–1.218)	0.592^†^
Moderate myopia	0.282 (0.193–0.411)	0.001^†^
Marked myopia	0.425 (0.292–0.620)	0.023^†^
After excluding the extent of myopia		
VCDR (per 0.1 larger)	2.595 (0.972–6.927)	0.001^‡^
Axial length (per mm longer)	0.796 (0.731–0.868)	0.008^‡^

MD = mean defect, OR = odds ratio, CI = confidence interval, VCDR = vertical cup-to-disk ratio.

^†^Candidate risk factors included: age, gender, right/left eye, central corneal thickness, BCVA (logMAR), vertical cup-to-disk ratio, baseline IOP, mean follow-up IOP, IOP reduction, IOP reduction percentage, initial MD, follow-up period, spherical equivalent, axial length, and the extents of myopia;

^‡^ Candidate risk factors included: age, gender, right/left eye; central corneal thickness, BCVA (logMAR), vertical cup-to-disk ratio, baseline IOP, mean follow-up IOP, IOP reduction, IOP reduction percentage, initial MD, follow-up period, spherical equivalent, and axial length

When logistic regression was repeated, excluding the extent of myopia, axial length (*p* = 0.008; OR = 0.796) and VCDR (*p* = 0.001; OR = 2.595 per 0.1 unit) showed significant importance (Table 2). Axial length was negatively associated with fast progression. We further compared the impacts of different degrees of axial length on VF progression, adjusting for age, gender, right/left eye, baseline IOP, mean follow-up IOP, BCVA (logMAR), CCT, VCDR, initial MD, and follow-up period (Table 3). Eyes with axial length ≤23 mm were set as a reference, those with axial length >24 mm among our patients exhibited a smaller chance for fast VF progression. Longer the axial length, smaller the OR values was noted for eyes with axial length >24 mm.

**Table 3:** Comparison of the impacts of different degrees of axial length on fast progression (MD slope >0.22 dB/y).

	OR (95%CI)	*p* Value
Axial length ≤23mm	Reference	Not available
Axial length >23 mm and ≤24 mm	0.334 (0.187–0.596)	0.059
Axial length >24 mm and ≤25 mm	0.309 (0.172–0.553)	0.044
Axial length >25 mm and ≤26 mm	0.266 (0.151–0.468)	0.019
Axial length >26 mm	0.260 (0.147–0.459)	0.018
Age at diagnosis (year)	1.018 (1.008–1.028)	0.077
Gender (male)	1.068 (0.808–1.412)	0.812
Right eye (left eye)	0.873 (0.664–1.147)	0.618
BCVA (logMAR)	2.472 (0.600–10.176)	0.522
CCT (um)	1.006 (1.002–1.010)	0.186
Vertical cup-to-disk ratio	16.432 (5.191–51.987)	0.015
Baseline IOP (mmHg)	1.031 (1.001–1.063)	0.312
Mean follow-up IOP (mmHg)	0.968 (0.901–1.041)	0.650
Initial MD (decibels)	1.025 (0.988–1.064)	0.505
Follow-up period (year)	1.043 (0.993–1.095)	0.394

MD = mean defect, OR = odds ratio, CI = confidence interval, BCVA (logMAR) = logarithm of the minimum angle of resolution of best-corrected visual acuity, CCT = central corneal thickness, IOP = intraocular pressure. The multivariate logistic regression analysis was performed after adjusting for age, gender, right/left eye, central corneal thickness, BCVA (logMAR), vertical cup-to-disk ratio, initial IOP, follow-up IOP, initial MD, and follow-up period

**Fig 1:**
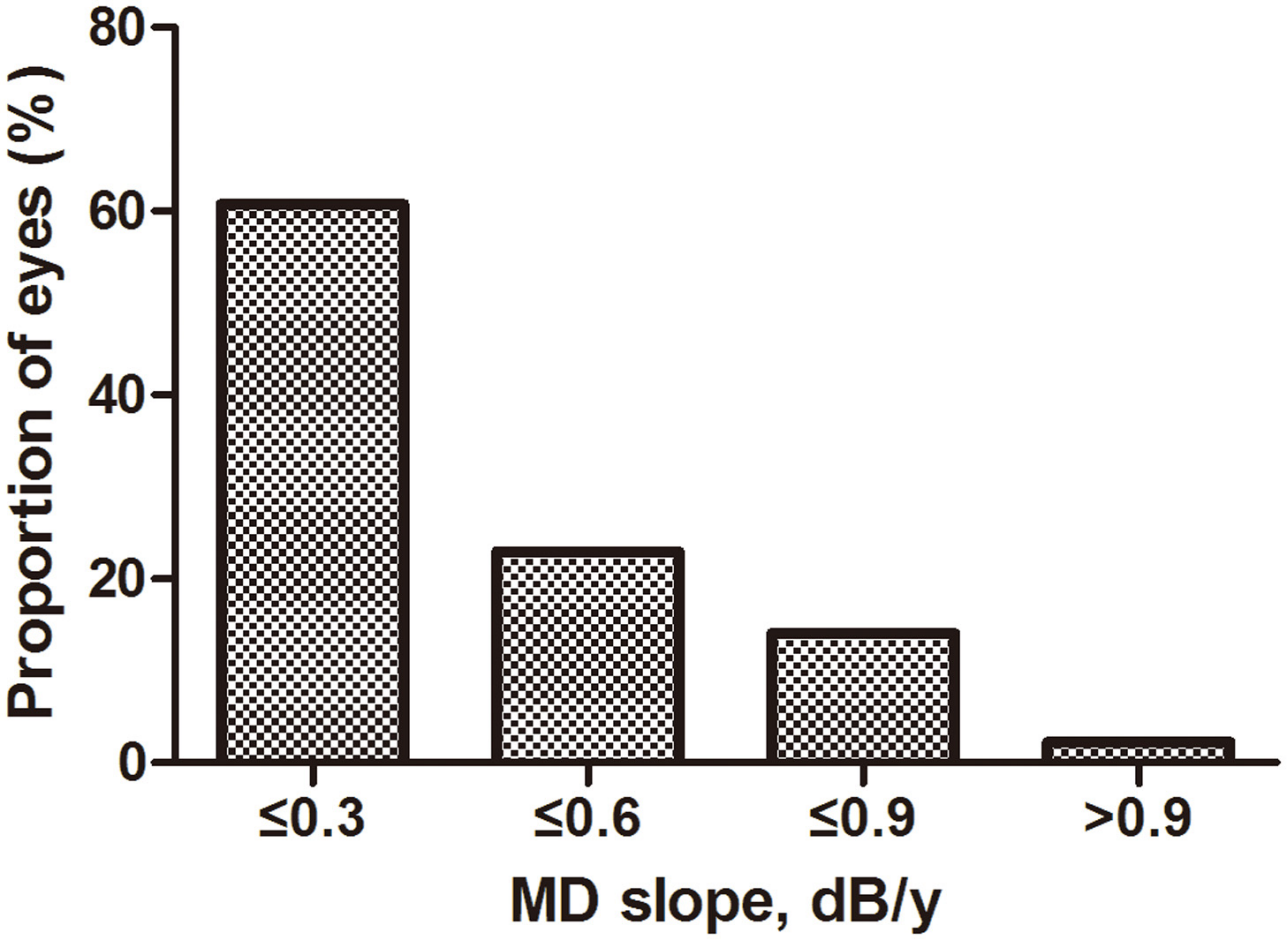
A distribution of the mean defect slope in the study. The mean value and the median of the mean defect slope are 0.284 ± 0.314 dB/y and 0.215dB/y, respectively. Patients identified as fast progression at the cutoff threshold of 0.30 dB/y were observed in 39.26% (106/270) of the study population.

According to the progression rate previously reported, 0.30 dB/y was also set to cutoff fast progression and slow progression. Date showed that 39.26% (106/270) of the study patients were classified as fast progression (Fig 1). The results of the logistic regression analysis were in accordance with the former ones (Table 4).

**Table 4:** Logistic regression analysis of factors associated with fast progression (MD slope >0.30 dB/y).

	OR (95% CI)	*p* Value
VCDR (per 0.1 larger)	1.979 (0.699–5.598)	0.004^†^
The extent of myopia		0.008^†^
Non-myopia	Reference	Not available
Mild myopia	1.098(0.729–1.655)	0.819^†^
Moderate myopia	0.387 (0.267–0.562)	0.011^†^
Marked myopia	0.469 (0.324–0.678)	0.040^†^
After excluding the extent of myopia		
VCDR (per 0.1 larger)	2.584 (0.931–7.174)	0.001^‡^
Axial length (per mm longer)	0.817 (0.749–0.891)	0.020^‡^

MD = mean defect, OR = odds ratio, CI = confidence interval, VCDR = vertical cup-to-disk ratio.

^†^Candidate risk factors included: age, gender, right/left eye, central corneal thickness, BCVA (logMAR), vertical cup-to-disk ratio, baseline IOP, mean follow-up IOP, IOP reduction, IOP reduction percentage, initial MD, follow-up period, spherical equivalent, axial length, and the extents of myopia;

^‡^Candidate risk factors included: age, gender, right/left eye; central corneal thickness, BCVA (logMAR), vertical cup-to-disk ratio, baseline IOP, mean follow-up IOP, IOP reduction, IOP reduction percentage, initial MD, follow-up period, spherical equivalent, and axial length

## Discussion

In this study, we set a threshold for fast/slow progression based on the median of the MD slope [15] or previously reported values [15,16]. Considering the difference of MD between Octopus (mean defect) and Humphrey perimetry (mean deviation) [17], 0.22 dB/y and 0.30 dB/y of Octopus may be comparable to the previous MD slope of Humphrey [11,15,16]. We found that VCDR and the extent of myopia were associated with fast/slow progression of VF. Compared to non-myopia, POAG patients with moderate and marked myopia were prone to slower deterioration. In addition, the negative association between axial length and fast progression supported the findings on myopia extent. Longer axial length was associated with a smaller chance for VF worsening.

IOP has been the longstanding focus of glaucoma field, even in normal-tension glaucoma (NTG). However, in accordance with the results of this study, some investigations similarly did not demonstrate such a relationship [9,18,19]. The suggestive coexistence of IOP-dependent and IOP-independent factors may be especially obvious in patients with lower IOPs. In the present study, 43.70% (118 out of 270) of the patients had a baseline IOP less than or equal to 21 mmHg, and most of the patients were relatively well controlled with IOP reductions of more than 30% under medical treatment (without surgeries). It is also possible that progression rate relates not to the absolute IOP level, but to the amount that IOP exceeds the damage threshold in each individual. Therefore, it was not surprising that IOP-independent factors impacted VF progression among these patients.

Estimation of CDR is the most widely used clinical method to assess glaucomatous damage. Subjective assessment of VCDR is also one of the best predictors of VF in glaucoma suspects [20] and in patients who were not severely impaired [19,21]. In the present study, VCDR was the most consistent risk factor for future VF loss in our patients. In contrast, other literatures, including the Advanced Glaucoma Intervention Study [22], did not suggest an important role for CDR. As the progression rate of VF damage may vary with glaucoma severity, we speculate that structural damage might be less evident than functional damage in advanced stages of the disease. Detection of VF progression may be easier in seriously deteriorated eyes.

The association between myopia and glaucoma progression is not thoroughly understood. Some researchers have claimed that severe myopia, especially pathological myopia, is often accompanied by thinner parapapillary sclera and lamina cribrosa [23] and an increased susceptibility to VF deterioration [12,21]. Others failed to find that myopia had a significant impact on VF progression in their clinical investigations [2,9–11]. We excluded severe myopic eyes with spherical equivalent more than -9.0 D in the present study due to the weak conformity in VF tests and the potential confounding influence of myopic VF deterioration. The results also demonstrated lack of a significant correlation between spherical equivalent and VF progression. Interestingly, moderate and marked myopia tended to preserve visual function as the conditions seemed less likely to have fast progression. These findings were consistent with the results of two Japanese studies that for non-high myopic eyes (<-8 D), mild myopia may be a risk factor for glaucomatous VF prognosis [13,14]. However, it is unclear whether this outcome was attributable to alterations in anatomic structures or other factors.

Axial length is one of the major factors contributing to myopia. However, axial length measurements have not commonly been obtained in previous studies of POAG. With the exception that axial myopia in the Meiktila Eye Study and the Singapore Malay Eye Study was a risk factor for having POAG [7,8]. Our statistical analysis indicated that longer axial length (>24 mm) may alleviate the threat for fast VF progression when compared to axial length ≤23 mm. The consistent, negative association of axial length with VF progression confirmed the aforementioned predictive value of myopia. However, differences should be noted from pathological myopia, where the weakened structure might be inclined to experience a faster progression rate [23].

The pathophysiological mechanism of axial myopia impacting VF prognosis is still unknown. One explanation may be that changes in ocular size may affect the acrophase and fluctuation of the circadian IOP. Longer axial length may manifest smaller eye-ball deformation for every 1 mm Hg increase in IOP. Jeong et al. [24] reported a negative correlation between nocturnal habitual-position IOP elevation and axial length. Compared to patients with mild myopia and non-myopia, those in the myopic group (<-3.0 D and axial length >24 mm) had a lower acrophase and a smaller range of 24-hour IOP fluctuation. The resultant fewer cumulative IOP insults and smaller lamina deformation might prevent rapid optic nerve injury.

Another possible explanation is that myopic glaucoma patients may experience biomechanical changes in the lamina cribrosa and peripapillary sclera during the disease process. Studies have validated the crucial impact of the biomechanical environment of optic nerve and peripapillary sclera [25], and indicated sclera remodelling of the posterior sclera and lamina cribrosa [26,27] in glaucoma eyes. The stiffer structure could possibly retard further glaucoma damage by constraining deformation of the sclera canal. This lends weight to our speculation that more extent of the non-pathological myopia may somehow be connected to more sclera remodeling. A stiffer sclera may be resistant to glaucoma damage, as suggested by longer and wider eyes in mice [28].

Several aspects limited our study. First, this study had relatively strict inclusion/exclusion criteria, which may have skewed the distribution of subjects. Second, we did not include other determinant factors of myopia, such as corneal curvature, crystal degree, which should be assessed in future studies. Third, certain risk factors reported by others, such as disk haemorrhage, blood pressure, and migraine, were not included. Additionally, it is also possible that a small proportion of subjects with increased primary macrocups may have been misclassified as glaucomatous. However, experienced ophthalmologists and patient observation of more than 3 years may eliminate the misdiagnosis to the utmost.

In conclusion, baseline VCDR and axial myopia were significant prognostic factors for glaucoma progression. Axial length could, in some way, signal a smaller risk for VF deterioration in these POAG patients.
